# Deregulation of circ_003912 contributes to pathogenesis of erosive oral lichen planus by via sponging microRNA-123, -647 and -31 and upregulating FOXP3

**DOI:** 10.1186/s10020-021-00382-4

**Published:** 2021-10-20

**Authors:** Zhen Huang, Fen Liu, Wenjuan Wang, Shaobo Ouyang, Ting Sang, Zikun Huang, Lan Liao, Jun Wu

**Affiliations:** 1 Jiangxi Provincial Key Laboratory of Oral Biomedicine, Department of Orthodontics, the Affiliated Stomatological Hospital of Nanchang University, Nanchang, 330006 China; 2 Jiangxi Provincial Key Laboratory of Oral Biomedicine, Department of Oral Prosthodontics, the Affiliated Stomatological Hospital of Nanchang University, Nanchang, 330006 China; 3 Clinical Laboratory Center, the First Affiliated Hospital of Nanchang University, No.49 Fuzhou Road, Nanchang, 330006 Jiangxi China

**Keywords:** Oral lichen planus, CD4+ Treg cells, FOXP3, miR-146a, circRNA, TRAF6

## Abstract

**Background:**

The FOXP3/miR-146a/NF-κB axis was previously reported to modulate the induction and function of CD4+ Treg cells to alleviate oral lichen planus. Also, other signaling pathways including microRNA-155-IFN-γ loop and FOXP3/miR-146a/TRAF6 pathways were reported to be involved in the pathogenesis of oral lichen planus. In this study, we aimed to investigate the molecular mechanism underlying the pathogenesis of EOLP.

**Method:**

CircRNA microarray was used to observe the expression of candidate circRNAs in CD4+ T-cells collected from different groups. Real-time PCR and Western blot were conducted to observe the changes in the expression of different miRNAs, mRNAs and proteins. Flow cytometry was performed to compare the counts of Treg cells in the HC and EOLP groups, and ELISA was performed to evaluate the changes in the expression of inflammatory cytokines.

**Result:**

No obvious differences were seen between the HC and EOLP groups in terms of age and gender. Among all candidate circRNAs, the expression of circ_003912 was most dramatically elevated in CD4+ T-cells collected from the EOLP group. The levels of miR-1231, miR-31, miR-647, FOXP3 mRNA and miR-146a were decreased while the expression of TRAF6 mRNA was increased in CD4+ T-cells collected from the EOLP group. The count of Treg cells in the EOLP group was dramatically increased. The levels of inflammatory cytokines including IL-4 IFN-γ, IL-10 and IL-2 were influenced by the presence of circ_003912. In CD4+ T-cells in the EOLP group, the levels of IL-4 and IL-10 were decreased while the levels of IFN-γ and IL-2 were increased. The presence of miR-1231, miR-31 and miR-647 all obviously inhibited the expression of circ_003912, which was validated to sponge the expression of above miRNAs. Also, FOXP3 mRNA was proved to be targeted by miR-1231, miR-31 and miR-647. Transfection of circ_003912 up-regulated the expression of circ_003912, miR-146a and FOXP3 mRNA/protein while down-regulating the expression of miR-1231, miR-31, miR-647, and TRAF6 mRNA/protein. The levels of inflammatory cytokines including IL-4 IFN-γ, IL-10 and IL-2 as well as the speed of cell proliferation were influenced by circ_003912.

**Conclusion:**

In this study, we investigated the molecular mechanisms underlying the pathogenesis of EOLP which involved the functioning of circ_003912. We first demonstrated that circ_003912 was up-regulated in CD4+ T-cells of the EOLP group. And miRNAs including miR-1231, miR-31 and miR-647 were sponged by circ_003912 and down-regulated in CD4+ T cells of the EOLP group, which subsequently up-regulated the expression of FOXP3 and miR-146a, and resulted in the inhibition of NF-kB.

**Supplementary Information:**

The online version contains supplementary material available at 10.1186/s10020-021-00382-4.

## Background

As a common diseases in oral mucosa, oral lichen planus (OLP) has been identified as a precancerous disease by the WHO (Scognamiglio 2017; McCartan and Healy 2008; Casparis et al. 2015). Currently, six types of OLP manifestations have been identified, i.e., papular lesions, reticular lesions, atrophic lesions, plaque lesions, erosive lesions as well as bullous lesions, while the erosive lesions are considered to be the ones most likely to progress into cancer (McCartan and Healy 2008; Casparis et al. 2015; Meij et al. 2007).

Circular RNA (circRNA) is a unique type of RNA involved in a number of pathological as well as physiological processes (Liu et al. 2017; Ng et al. 2017). Accumulating evidence has shown that several circRNAs can act as competing endogenous RNAs (ceRNAs) can act as diagnostic as well as prognostic biomarkers of medical conditions by inhibiting their target microRNAs (miRNAs) via miRNA sponging (Kulcheski et al. 2016; Rong et al. 2017).

As one of the forkhead-box/winged-helix transcription factors, FOXP3 is well-known to play a regulatory role in gene transcription in regulatory T cells (Hori et al. 2003). The ablation in the expression of Foxp3 can cause prostate hyperplasia in mice, indicating that Foxp3 can act as a tumor suppressor during the initiation of tumors (Wang et al. 2009). In addition, it was demonstrated that FOXP3 could suppress the proliferation of tumor cells in prostate cancer (Wang et al. 2009). Additionally, FOXP3 was shown to suppress the migration, proliferation as well as invasion of tumor cells in melanoma, breast cancer, glioblastoma as well as ovarian cancer (Liu et al. 2009; Zuo et al. 2007a,2007b; Zhang and Sun 2010; Tan et al. 2014). FOXP3 can also mediate the immune response as well as inflammation in the body by regulating Treg cells (Mohr et al. 2019). It was also found that the expression of FOXP3 was elevated significantly in tissues of OLP (Xie et al. 2014).

Fopx3 regulates the expression of miR-146 family members, such as miR-146b and miR-146a, in numerous diseases, such as prostate cancer, breast cancer, as well as T cell-mediated diseases (Lu et al. 2010; Liu et al. 2015). It was also shown that Foxp3 can bind to miR-146a promoter to significantly increase its transcriptional (Wang et al. 2018). MiR-146a can target TRAF6, a downstream factor of TLR4, to modulate NK-κB transcription to induce inflammatory responses and exert negative regulation on the immune functions (Varney et al. 2015). It was also found that TRAF6 expression was significantly increased in patients with LN (lupus nephritis), indicating that the reduced miR-146a expression and increased TRAF6 expression may contribute to the pathogenesis of LN (Zhu et al. 2017).

The family of tumour necrosis factor receptor (TNFR)-associated factors (TRAF) contains TRAF1–7 proteins, which are critical enzymatic adaptor proteins. Among them, TRAF2/3/5/6 plays a role as ubiquitin E3 ligases, while TRAF6 also plays an essential role in immune responses as well as tissue homoeostasis (Xie 2013; Lomaga et al. 1999; Naito et al. 2002). In a previous study, Muto et al. found that Traf6fl/fl Foxp3Cre+ mice were prone to develop lymphoproliferation, dermatitis, as well as immune pathologies. Nevertheless, the knockout of Tregs in these mice did not significantly suppress above pathologies, but the Tregs of TRAF6 knockout were unstable phenotypically (Ni et al. 2019; Muto et al. 2013). It was actually revealed that the elevated level of Tregs frequently seen in OLP was correlated to the theory that the malfunction of suppressing mechanisms mediated by Tregs is associated with OLP pathogenesis (Zhou et al. 2016).

The FOXP3/miR-146a/NF-κB axis was previously reported to modulate the induction and function of CD4+ Treg cells to alleviate oral lichen planus, while other signaling pathways including microRNA-155-IFN-γ loop and FOXP3/miR-146a/TRAF6 pathways are involved in the pathogenesis of oral lichen planus (Wang et al. 2018, 2019; Hu et al. 2015). In this study, we aimed to investigate the molecular mechanism underlying the pathogenesis of EOLP.

## Materials and methods

### Patient recruitment and grouping

In this study, a total of 65 EOLP patients were recruited into the EOLP group (N = 65) along with 70 healthy control subjects, who were included in the HC group (N = 70). The peripheral blood samples were collected from each subject in the two groups to isolate CD4+ T-cells using a CD4+ T Cell Isolation Kit (Miltenyi Biotec, Germany) following instruction provided by the manufacturer, and the expression of various target circRNAs in isolate CD4+ T-cells from each subject was assayed and compared by using circRNA microarray (Arraystar, Rockville, MD) following instruction provided by the manufacturer.

### Cell culture and transfection

The THP-1 cell line was obtained from ATCC (Manassas, VA) and maintained in a standard RPMI 1640 medium (Gibco, Thermo Fisher Scientific, Waltham, MA) supplemented with 100 IU/ml penicillin, 10% FBS, as well as 100 μg/ml streptomycin. The culture was carried out at 37 °C in a humidified tissue culture incubator containing 5% CO_2_ and 95% air. After the THP-1 cells reached 80% confluence, they were divided into different groups and received corresponding treatments, as shown below.

Cell model 1: THP-1 cells were randomly divided into different groups and transfected with miR-1204, miR-1231, miR-1248, miR-1253, miR-1290, miR-31, miR-433, miR-578, miR-609, miR-619, miR-647, and miR-767-3p, respectively. The transfection was carried out by using Fugene 6 HD (Promega, Madison, WI) in reference with the recommended transfection protocol provided on the manufacturer’s manual. At 48 h after the start of transfection, the cells were harvested for the analysis of target gene expression. Cell model 2: THP-1 cells were randomly divided into two groups, i.e., (1) NC group (THP-1 cells treated with PBS only); and (2) p-circ_003912 group (THP-1 cells transfected with p-circ_003912). The transfection was also carried out by using Fugene 6 HD and the cells were harvested after 48 h of transfection to analyze target gene expression. Cell model 3: THP-1 cells were randomly divided into two groups, i.e., (1) NC group (THP-1 cells treated with PBS only); and (2) circ_003912 siRNA group (THP-1 cells transfected with circ_003912 siRNA). The transfection was also carried out by using Fugene 6 HD and the cells were harvested after 48 h of transfection to analyze target gene expression.

### RNA isolation and real-time PCR

Real-time PCR was performed to observe the differences in the expression of several miRNAs or mRNAs potentially involved in the signaling of circ_003912. After the homogenization of collected cell and tissue samples, the RNA content of the samples was separated by using Trizol (Invitrogen, Carlsbad, CA) in reference with the recommended operating protocol provided on the manufacturer’s manual. In the next step, the quality as well as quantity of the extracted RNA samples was identified by using a Nano Drop ND-1000 spectrometer (NanoDrop, Wilmington, NJ) in reference with the recommended operating protocol provided on the manufacturer’s manual. Then, one-step real-time PCR was done by making use of a SYBR Green assay kit on a LightCycler 480 real-time PCR system (Roche Diagnostics, Indianapolis, IN) in reference with the recommended assay protocol provided on the manufacturer’s manual. Finally, the relative expression of circ_003912 (Forward: 5′-TGTTGTGGAAGAAGAGGGCAG-3′; Reverse: 5′-AAAGGCAGTCGCTTCATTCCT-3′), miR-1231 (Forward: 5′-TCTGGGCGGACAGCTG-3′; Reverse: 5′-GAACATGTCTGCGTATCTC-3′), miR-31 (Forward: 5′- GCAAGATGCTGGCATAG-3′; Reverse: 5′-GAACATGTCTGCGTATCTC-3′), miR-647 (Forward: 5′-TGGCTGCACTCACTTCC-3′; Reverse: 5′-GAACATGTCTGCGTATCTC-3′), FOXP3 mRNA (Forward: 5′-GGCACAATGTCTCCTCCAGAGA-3′; Reverse: 5′- CAGATGAAGCCTTGGTCAGTGC-3′), miR-146a (Forward: 5′-GAGAACTGAATTCCATGG-3′; Reverse: 5′-GAACATGTCTGCGTATCTC-3′), and TRAF6 mRNA (Forward: 5′-CAATGCCAGCGTCCCTTCCAAA-3′; Reverse: 5′- CCAAAGGACAGTTCTGGTCATGG-3′) in each sample was calculated by using the Ct method and normalized to that of the internal control β-actin.

### Vector construction, mutagenesis and luciferase assay

According to the results obtained from our computational analysis, putative miR-31, miR-1231 and miR-647 binding sites were found on the sequence of circ_003912, respectively. Therefore, in order to clarify the relationship between circ_003912 and miR-31, miR-1231, miR-647 and FOXP3 mRNA, i.e., circ_003912/miR-31, circ_003912/miR-1231, circ_003912/miR-647, miR-647/FOXP3, miR-1231/FOXP3, we carried out a luciferase assay in THP-1 cells. In brief, wild type sequences of circ_003912 containing the miR-31, miR-1231 and miR-647 binding sites, respectively, were cloned into pcDNA3.1 luciferase vectors (Promega, Madison, WI) in reference with the recommended cloning protocol provided on the manufacturer’s manual to generate wild type circ_003912 plasmids. At the same time, a Quick Change mutagenesis assay kit (Stratagene, San Diego, CA) was used in reference with the recommended assay protocol provided on the manufacturer’s manual to generate mutant type circ_003912 plasmids containing site-directed mutations in the putative miR-31, miR-1231 and miR-647 binding sites, respectively. In the next step, wild type and mutant type circ_003912 plasmids were co-transfected into THP-1 cells with miR-31, miR-1231 and miR-647 mimics, circ_003912 siRNA or a scramble control using Lipofectamine 2000 (Invitrogen, Carlsbad, CA) in reference with the recommended transfection protocol provided on the manufacturer’s manual. At 48 h post transfection, the luciferase activity of transfected THP-1 cells was measured by utilizing a Bright Glo Luciferase Gene Reporter Assay Kit (Promega, Madison, WI) in reference with the recommended assay protocol provided on the manufacturer’s manual. The reading of luciferase activity signals was done on a Veritas luminometer (Turner Biosystems, Atlanta, GA) in triplicates.

### Western blot analysis

Cells were pre-lysed in a NP40 lysis solution (pH 7.5) containing 150 mM of KCl, 50 mM of HEPES, 0.5% (v/v) of NP40, 1 mM of NaF, 2 mM of EDTA, and 0.5 mM of dithiothreitol. The concentrations of the proteins in various collected samples were measured by making use of a BCA assay kit (Thermo Fisher Scientific, Waltham, MA) in reference with the recommended assay protocol provided on the manufacturer’s manual. In the next step, 25 μg of total protein in each sample was separated on a 10% SDS-PAGE gel and transferred to a PVDF membrane, which was then blocked by using TBS-Tween containing 5% skim milk before being probed using primary anti-FOXP3 and anti-TRAF6 antibodies as well as suitable HRP-labeled secondary antibodies. All antibodies were purchased from Abcam (Cambridge, MA) and used in reference with the recommended incubation protocol provided on the manufacturer’s manual. Finally, the protein bands were developed by utilizing an enhanced chemiluminescence reagent (Thermo Fisher Scientific, Waltham, MA) in reference with the recommended assay protocol provided on the manufacturer’s manual and evaluated by using NIH ImageJ software to calculate the relative expression of FOXP3 and TRAF6 proteins normalized to the expression of β-actin in each sample.

### ELISA

The levels of IL-4, IFN-γ, IL-10, and IL-2 in collected peripheral blood samples were assayed by using commercially available enzyme-linked immunosorbent assay (ELISA) assay kits (NeoBioscience, Beijing, China) in reference with the recommended assay protocol provided on the manufacturer’s manual.

### Flow cytometry

The number of Treg cells in each collected sample was counted by using an Annexin V-FITC and propidium iodide staining solution (Thermo Fisher Scientific, Waltham, MA) in reference with the recommended assay protocol provided on the manufacturer’s manual. The Treg cells were isolated using a CD4+CD25+ Regulatory T Cell Isolation Kit (Miltenyi Biotec, Germany) following instruction provided by the manufacturer. The result reading was carried out in a FACS Calibur flow cytometer (BD, San Jose, CA).

### Statistical analysis

The statistical analysis was done by using SPSS software version 21.0 (SPSS, IBM, Armonk, NY). All results were expression as mean ± SD. All inter-group comparisons were done using one-way ANOVA and student’s *t*-test. The level of statistical significance was set to 0.05.

## Results

### General information of patients and healthy controls

In this study, a total of 65 EOLP patients were recruited into the EOLP group (N = 65) along with 70 healthy control subjects (HC group, N = 70). CD4+ T-cells were collected from the subjects in different groups for further analysis. As shown in Table 1, the physical and clinicopathological information of the two groups showed no obvious differences in terms of age and gender.

**Table 1:** Patient information of HC and EOLP groups

Characteristics	HC (N = 70)	EOLP (N = 65)	*P* value
Age (years)	45.1 ± 3.8	46.2 ± 3.6	0.943
Sex			0.541
Male	38	33	
Female	32	32	
Disease duration (month)	NA	24.7 ± 3.2	

### The expression of various circRNAs in CD4+ T-cells from different groups

By searching the literature, we studied the expression of various candidate circRNAs in CD4+ T-cells collected from EOLP patients or healthy controls. As shown in Table 2, the circRNA microarray demonstrated that 12 circRNAs were up-regulated while 9 circRNAs were down-regulated in EOLP patients. Among them, the expression of circ_003912 was most dramatically elevated in the CD4+ T-cells collected from the EOLP group. Therefore, we selected circ_003912 to study the molecular mechanisms underlying the pathogenesis of EOLP.

**Table 2:** Differentially expressed  circRNAs identified in EOLP patients

MiRNA	HC group (N = 70)	EOLP group (N = 65)	Fold change
Up-regulated circRNAs			
hsa_circ_0000788	2.3137	4.3826	1.8942
hsa_circ_043621	0.6938	1.3419	1.9341
hsa_circ_104075	1.5513	3.4696	2.2366
hsa_circ_003794	2.6472	6.7215	2.5391
hsa_circ_0058794	3.0694	8.8755	2.8916
hsa_circ_0076092	0.5963	1.7683	2.9655
hsa_circ_001175	0.2941	0.9049	3.0768
hsa_circ_101755	1.8324	5.9128	3.2268
hsa_circ_0013058	1.5618	5.2655	3.3714
hsa_circ_100438	0.6453	2.3186	3.5931
hsa_circ_0092285	0.2288	0.8409	3.6753
hsa_circ_003912	0.1577	0.6676	4.2334
Down-regulated circRNAs			
hsa_circ_0012919	1.3283	0.3261	0.2455
hsa_circ_002059	5.1368	1.6905	0.3291
hsa_circ_100797	2.4878	0.8675	0.3487
hsa_circ_0067934	2.3199	0.9252	0.3988
hsa_circ_102459	1.8642	0.8175	0.4385
hsa_circ_0007991	0.6871	0.3286	0.4782
hsa_circ_102049	0.7634	0.368	0.4821
hsa_circ_001283	0.5517	0.2768	0.5017
hsa_circ_0005699	4.3981	2.2646	0.5149

### The expression of several miRNAs, mRNAs and circ_003912 in CD4+ T-cells collected from different groups

**Fig. 1:**
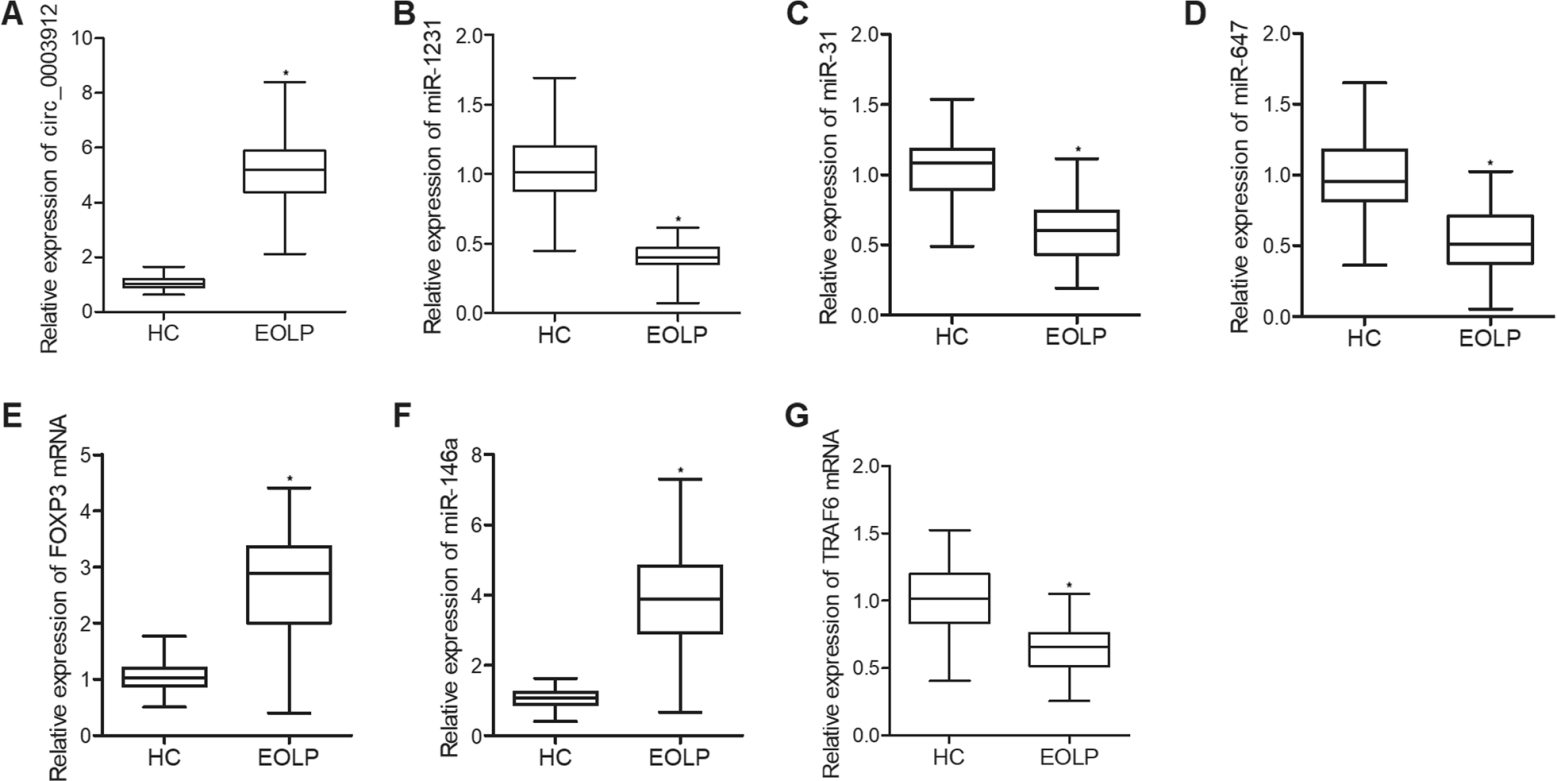
The expression of several miRNAs, mRNAs and circ_003912 in CD4+ T-cells collected from different groups (*P value < 0.05 compared with HC group, student’s *t*-test). **A** relative expression of circ_003912 in CD4+ T-cells collected from different groups; **B** relative expression of miR-1231 in CD4+ T-cells collected from different groups; **C** relative expression of miR-31 in CD4+ T-cells collected from different groups; **D** relative expression of miR-647 in CD4+ T-cells collected from different groups; **E** relative expression of FOXP3 mRNA in CD4+ T-cells collected from different groups; **F** relative expression of miR-146a in CD4+ T-cells collected from different groups; **G** relative expression of TRAF6 mRNA in CD4+ T-cells collected from different groups

Real-time PCR was performed to observe the differences in the expression of several miRNAs or mRNAs potentially involved in the signaling of circ_003912. As shown in Fig. 1, the level of circ_003912 (Fig. 1A) was evidently increased in CD4+ T-cells collected from the EOLP group, and the levels of miR-1231 (Fig. 1B), miR-31 (Fig. 1C) and miR-647 (Fig. 1D) were all decreased in CD4+ T-cells collected from the EOLP group. The expression of FOXP3 mRNA (Fig. 1E) and miR-146a (Fig. 1F) was promoted while the TRAF6 mRNA expression (Fig. 1G) was inhibited in CD4+ T-cells collected from the EOLP group.

### Levels of Treg cells and inflammatory cytokines in different groups

**Fig. 2:**
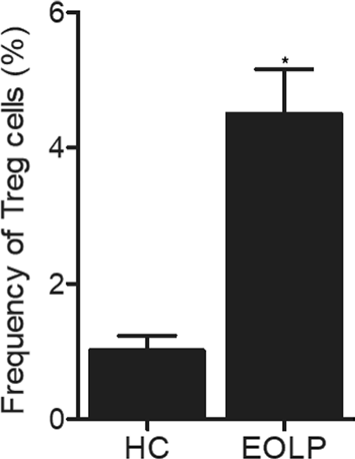
Quantification of flow cytometry count of Treg cells in the HC and EOLP groups (*P value < 0.05 compared with HC group, student’s *t*-test)

**Fig. 3:**
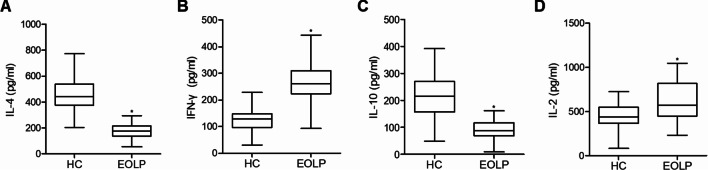
Levels of inflammatory cytokines in different groups (*P value < 0.05 compared with HC group, student’s *t*-test). **A** level of IL-4 in different groups; **B** level of IFN-γ in different groups; **C** level of IL-10 in different groups; **D** level of IL-2 in different groups

As shown in Fig. 2, the flow cytometry assay indicated that the count of Treg cells in the EOLP group was dramatically increased as compared with that in the control group. Also, IL-4 (Fig. 3A) and IL-10 (Fig. 3C) expression was decreased while IFN-γ (Fig. 3B) and IL-2 (Fig. 3D) expression was increased in the EOLP group.

### Presence of several miRNAs influenced the expression of circ_003912

**Fig.4:**
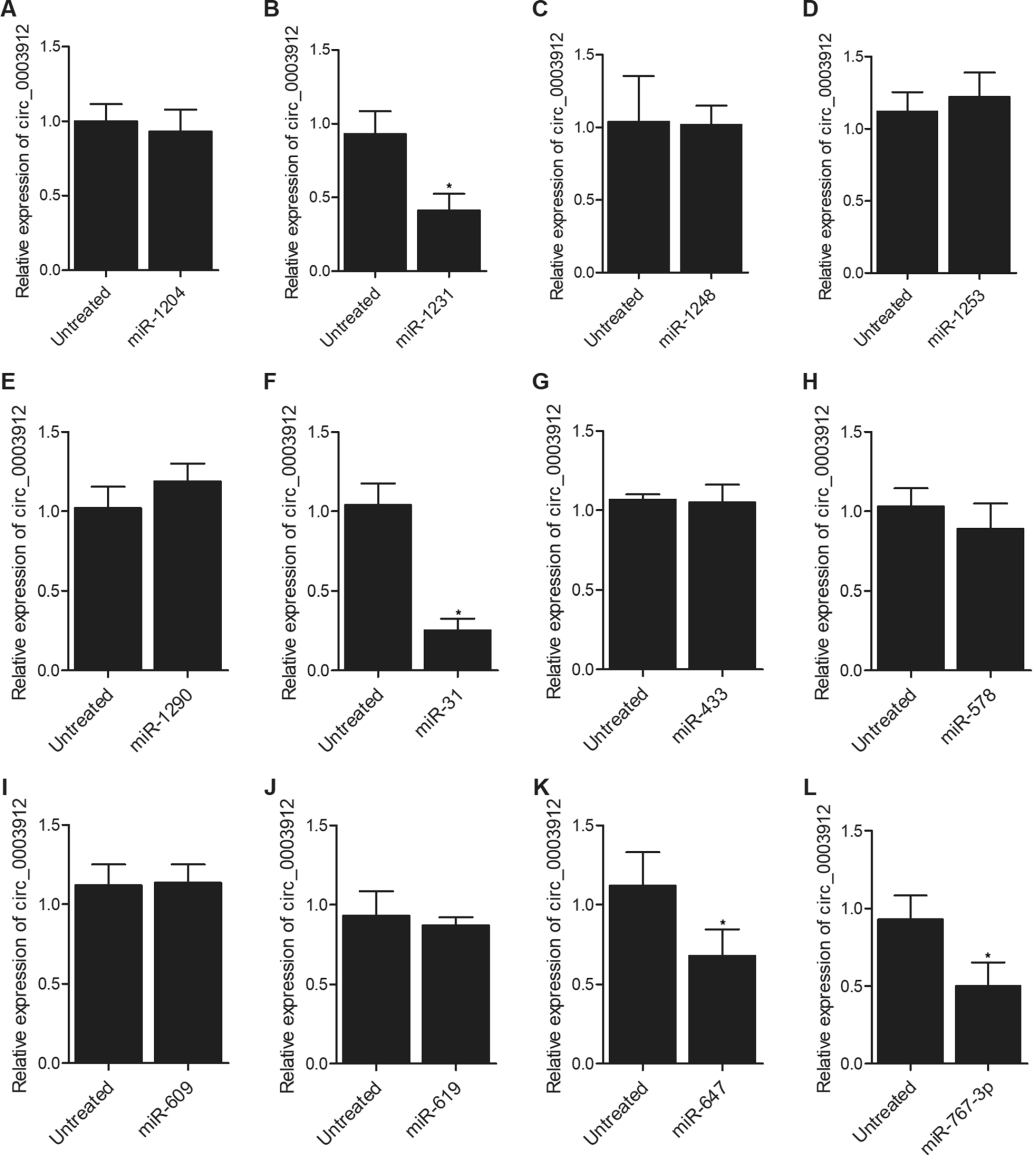
Presence of several miRNAs influenced the expression of circ_003912 (*P value < 0.05 compared with untreated group, student’s *t*-test). **A** transfection of miR-1204 exhibited no effect upon the expression of circ_003912; **B** transfection of miR-1231 obviously inhibited the expression of circ_003912; **C** transfection of miR-1248 exhibited no effect upon the expression of circ_003912; **D** transfection of miR-1253 exhibited no effect upon the expression of circ_003912; **E** transfection of miR-1290 exhibited no effect upon the expression of circ_003912; **F** transfection of miR-31 obviously inhibited the expression of circ_003912; **G** transfection of miR-433 exhibited no effect upon the expression of circ_003912; **H** transfection of miR-578 exhibited no effect upon the expression of circ_003912; **I** transfection of miR-609 exhibited no effect upon the expression of circ_003912; **J** transfection of miR-619 exhibited no effect upon the expression of circ_003912; **K** transfection of miR-647 obviously inhibited the expression of circ_003912; **L** transfection of miR-767-3p obviously inhibited the expression of circ_003912

Several candidate miRNAs were selected to transfect THP-1 cells to observe their effects on the expression of circ_003912. As shown in Fig. 4, the presence of miR-1204 (Fig. 4A), miR-1248 (Fig. 4C), miR-1253 (Fig. 4D), miR-1290 (Fig. 4E), miR-433 (Fig. 4G), miR-578 (Fig. 4H), miR-609 (Fig. 4I) and miR-619 (Fig. 4J) did not affect the expression of circ_003912 in THP-1 cells, but the presence of miR-1231 (Fig. 4B), miR-31 (Fig. 4F), miR-647 (Fig. 4K) and miR-767-3p (Fig. 4L) all obviously inhibited the expression of circ_003912 in THP-1 cells, suggesting the presence of potential interactions among miR-1231, miR-31, miR-647 and circ_003912.

### MiR-31, miR-1231 and miR-647 targeted the expression of circ_003912 and FOXP3

**Fig.5:**
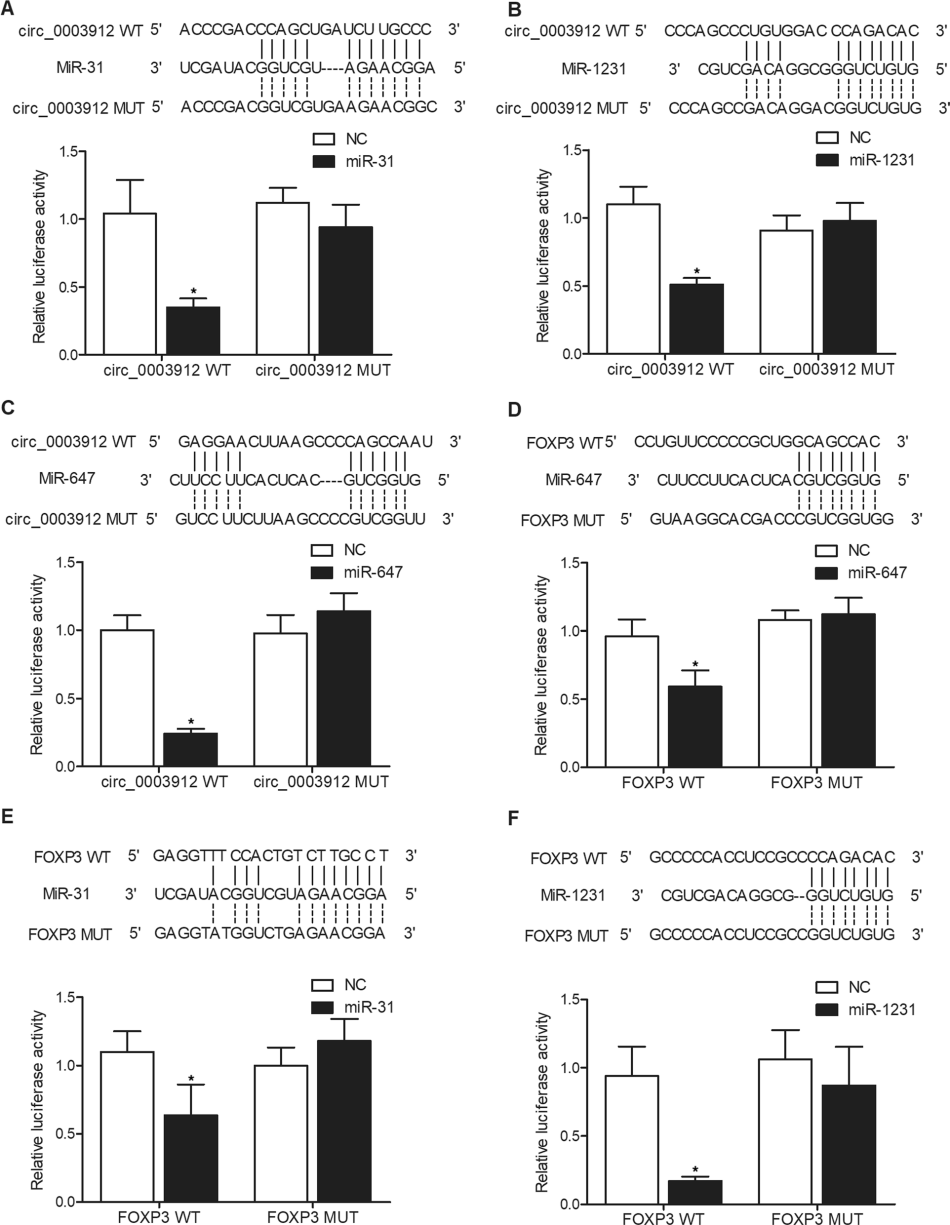
MiR-31, miR-1231 and miR-647 targeted the expression of circ_003912 and FOXP3 (*P value < 0.05 compared with NC group, one-way ANOVA). **A** computational analysis and luciferase assay indicated that miR-31 was sponged by circ_003912; **B** computational analysis and luciferase assay indicated that miR-1231 was sponged by circ_003912; **C** computational analysis and luciferase assay indicated that miR-647 was sponged by circ_003912; **D** computational analysis and luciferase assay indicated that FOXP3 mRNA was targeted by miR-647; **E** computational analysis and luciferase assay indicated that FOXP3 mRNA was targeted by miR-31; **F** computational analysis and luciferase assay indicated that FOXP3 mRNA was targeted by miR-1231

As indicated by the results of a computational analysis, putative miR-31 (Fig. 5A), miR-1231 (Fig. 5B) and miR-647 (Fig. 5C) binding sites were found on circ_003912, respectively. Luciferase assay was conducted in THP-1 cells to define the relationship between circ_003912 and miR-31, miR-1231 and miR-647. As shown in Fig. 5 and compared with other groups, the luciferase activity of wild-type circ_003912 was the lowest in THP-1 cells transfected with miR-31 (Fig. 5A), miR-1231 (Fig. 5B) or miR-647 (Fig. 5C), indicating that these miRNAs were all sponged by circ_003912. Meanwhile, the computational analysis also identified miR-647 (Fig. 5D), miR-31 (Fig. 5E) and miR-1231 (Fig. 5F) binding sites in the 3′UTR of FOXP3 mRNA, and the luciferase activity was significantly decreased in PTNECE01 cells transfected with wild-type FOXP3 mRNA in the presence of miR-647 (Fig. 5D), miR-31 (Fig. 5E) and miR-1231 (Fig. 5F), respectively. Therefore, it could be concluded that FOXP3 was targeted by miR-674, miR-31 and miR-1231, respectively.

### Signaling pathways involved in the pathogenesis of EOLP

**Fig. 6:**
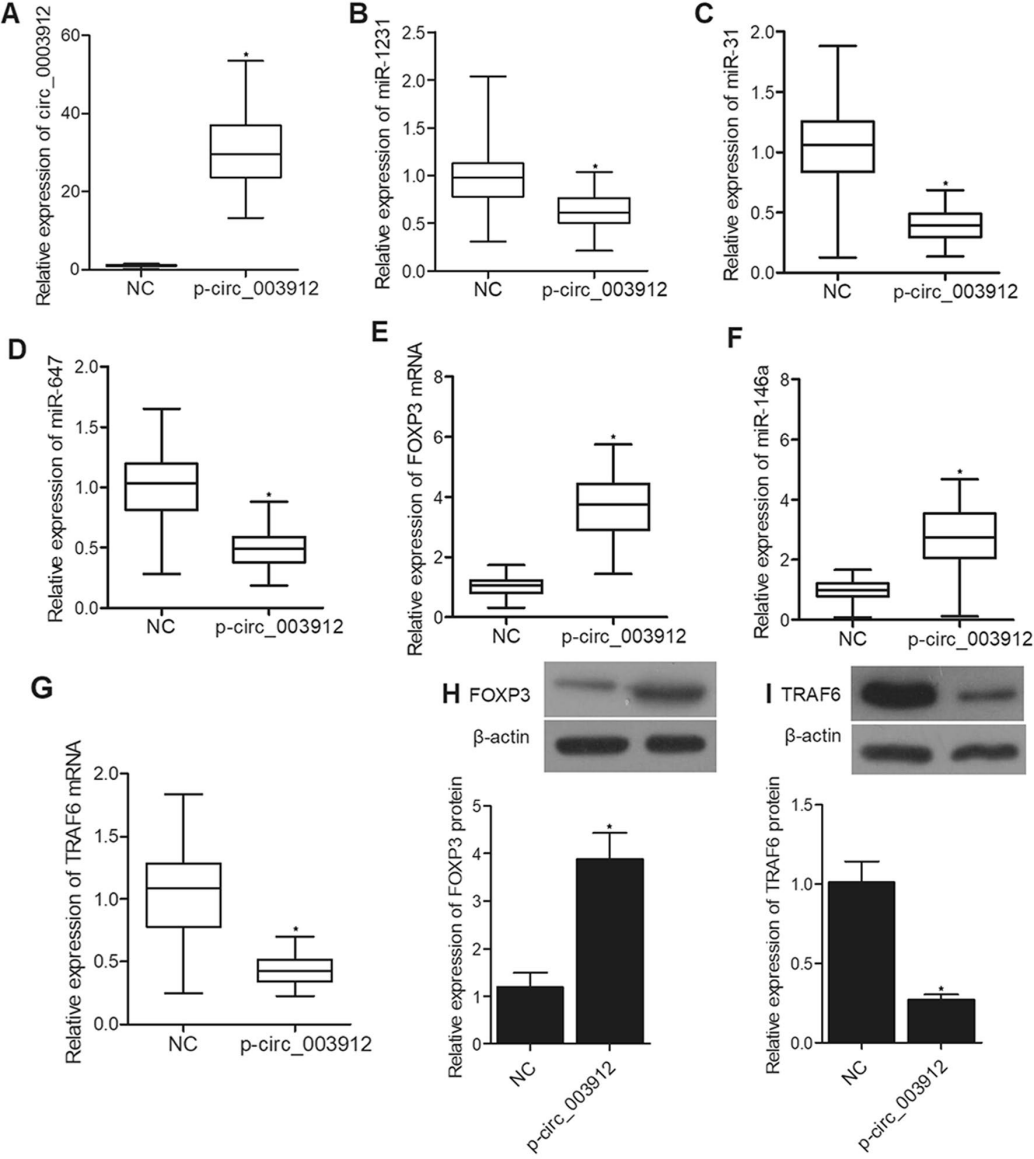
Transfection of circ_003912 influenced the expression of downstream miRNAs and mRNAs (*P value < 0.05 compared with NC group, student’s *t*-test). **A** transfection of circ_003912 up-regulated the expression of circ_003912; **B** transfection of circ_003912 down-regulated the expression of miR-1231; **C** transfection of circ_003912 down-regulated the expression of miR-31; **D** transfection of circ_003912 down-regulated the expression of miR-647; **E** transfection of circ_003912 up-regulated the expression of FOXP3 mRNA; **F** transfection of circ_003912 up-regulated the expression of miR-146a; **G** transfection of circ_003912 down-regulated the expression of TRAF6 mRNA; **H** transfection of circ_003912 up-regulated the expression of FOXP3 protein; **I** transfection of circ_003912 down-regulated the expression of TRAF6 protein

**Fig. 7:**
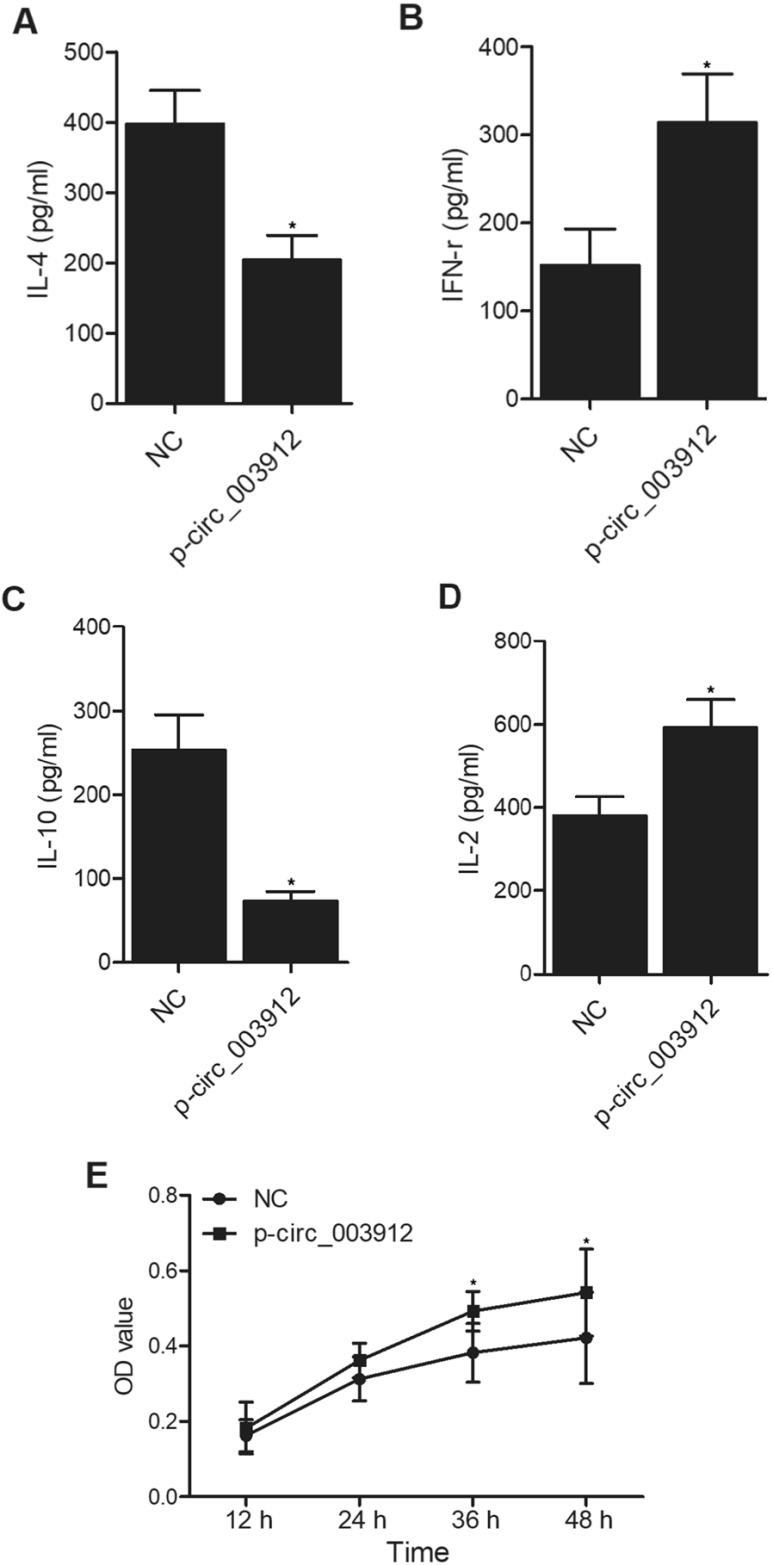
Transfection of circ_003912 influenced the levels of inflammatory cytokines and cell proliferation of THP-1 cells (*P value < 0.05 compared with NC group, student’s *t*-test). **A** transfection of circ_003912 decreased the level of IL-4 in cell supernatants of THP-1 cells; **B** transfection of circ_003912 increased the level of IFN-r in cell supernatants of THP-1 cells; **C** transfection of circ_003912 decreased the level of IL-10 in cell supernatants of THP-1 cells; **D** transfection of circ_003912 increased the level of IL-2 in cell supernatants of THP-1 cells; **E** transfection of circ_003912 increased the proliferation of THP-1 cells

**Fig. 8:**
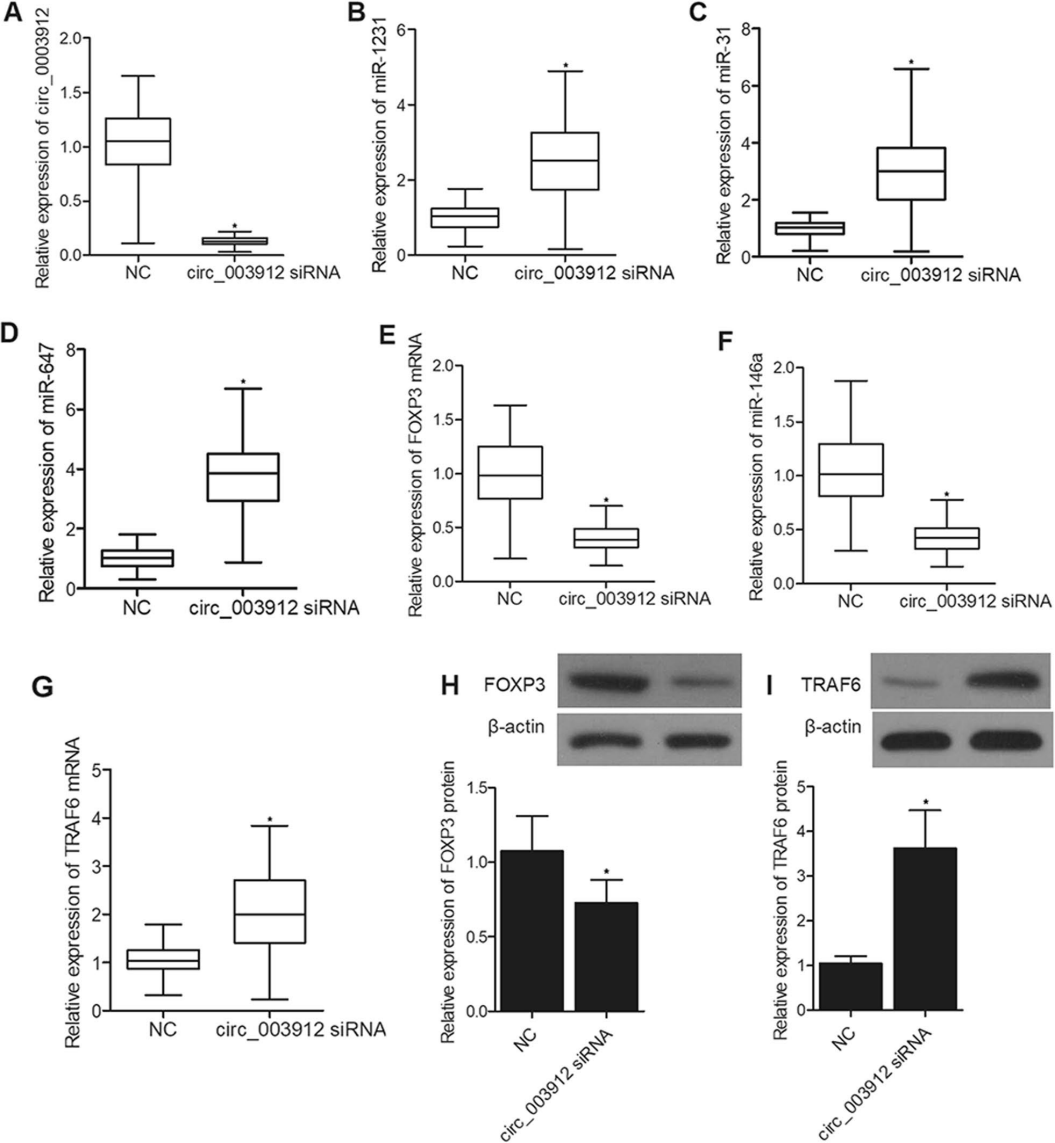
Transfection of circ_003912 siRNA influenced the expression of downstream miRNAs and mRNAs (*P value < 0.05 compared with NC group, student’s *t*-test). **A** transfection of circ_003912 siRNA down-regulated the expression of circ_003912; **B** transfection of circ_003912 siRNA up-regulated the expression of miR-1231; **C** transfection of circ_003912 siRNA up-regulated the expression of miR-31; **D** transfection of circ_003912 siRNA up-regulated the expression of miR-647; **E** transfection of circ_003912 siRNA down-regulated the expression of FOXP3 mRNA; **F** transfection of circ_003912 siRNA down-regulated the expression of miR-146a; **G** transfection of circ_003912 siRNA up-regulated the expression of TRAF6 mRNA; **H** transfection of circ_003912 siRNA down-regulated the expression of FOXP3 protein; **I** transfection of circ_003912 siRNA up-regulated the expression of TRAF6 protein

**Fig. 9:**
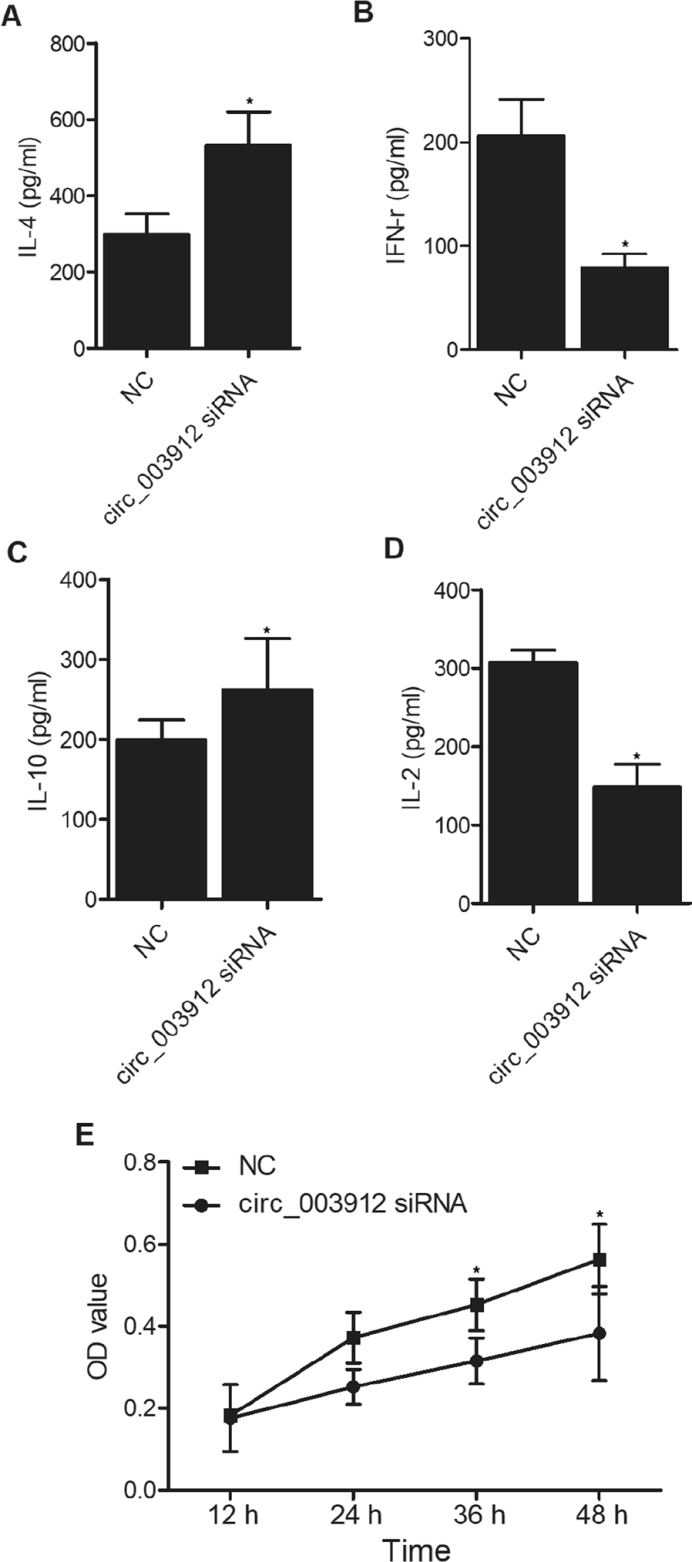
Transfection of circ_003912 siRNA influenced the levels of inflammatory cytokines and cell proliferation of THP-1 cells (*P value < 0.05 compared with NC group; student’s *t*-test). **A** transfection of circ_003912 siRNA increased the level of IL-4 in cell supernatants of THP-1 cells; **B** transfection of circ_003912 siRNA decreased the level of IFN-r in cell supernatants of THP-1 cells; **C** transfection of circ_003912 siRNA increased the level of IL-10 in cell supernatants of THP-1 cells; **D** transfection of circ_003912 siRNA decreased the level of IL-2 in cell supernatants of THP-1 cells; **E** transfection of circ_003912 siRNA decreased the proliferation of THP-1 cells

To investigate the underlying molecular mechanisms, THP-1 cells were transfected with plasmids carrying circ_003912. As shown in Fig. 6, the presence of circ_003912 not only significantly up-regulated the expression levels of circ_003912 (Fig. 6A), FOXP3 mRNA (Fig. 6E), FOXP3 protein (Fig. 6H) and miR-146a (Fig. 6F), but also obviously down-regulated the expression levels of miR-1231 (Fig. 6B), miR-31 (Fig. 6C), miR-647 (Fig. 6D), TRAF6 mRNA (Fig. 6G) and TRAF6 protein (Fig. 6I). Also, the expression levels of IL-4 (Fig. 7A) and IL-10 (Fig. 7C) were inhibited while the expression levels of IFN-r (Fig. 7B) and IL-2 (Fig. 7D) were promoted in the p-circ_003912 group. CKK8 assay also validated that circ_003912 promoted cell proliferation (Fig. 7E). On the contrary, when THP-1 cells were transfected with circ_003912 siRNA, the levels of circ_003912 (Fig. 8A), FOXP3 mRNA (Fig. 8E), FOXP3 protein (Fig. 8H), miR-146a (Fig. 8F), IFN-r (Fig. 9B) and IL-2 (Fig. 9D) were all decreased while the levels of miR-1231 (Fig. 8B), miR-31 (Fig. 8C), miR-647 (Fig. 8D), TRAF6 mRNA (Fig. 8G), TRAF6 protein (Fig. 8I), IL-4 (Fig. 9A) and IL-10 (Fig. 9C) were all increased. Also, the proliferation of THP-1 cells (Fig. 9E) was inhibited by the transfection of circ_003912 siRNA.

## Discussion

In this study, we performed microarray studies to determine the circRNAs differentially expressed between the CD4+ T cells collected from the control and ELOP groups. Among all candidate circRNAs, the expression of circ_003912 was most dramatically elevated in CD4+ T-cells collected from the EOLP group. The levels of miR-1231, miR-31, miR-647, FOXP3 mRNA and miR-146a were decreased while the expression of TRAF6 mRNA was increased in CD4+ T-cells collected from the EOLP group.

As the sole member in a miRNA ‘seed family’ that exists in both Drosophila and vertebrates, miR-31 can regulate the differentiation of keratinocytes by suppressing hypoxia-inducible factor 1 (Peng et al. 2012; Valastyan and Weinberg 2010). In addition, different from other subsets of T-cells, miR-31 is suppressed in Treg cells in humans 21, suggesting that the expression of miR-31 is targeted and reduced in Treg cells (Zhang et al. 2015). Previously, modified miR-31 expression was actually reported in tissues samples of OLP, and a potential relationship between the premalignant transformation of OLP and the levels of miR-31 expression was pointed out (Zhang et al. 2012; Gassling et al. 2013). Similar results were also found in patients of OSCC and dysplastic OLP (Mehdipour et al. 2018; Lu et al. 2014; Wang et al. 2014). In this study, we found that the count of Treg cells was dramatically increased in the EOLP group. Also, the expression of IL-4 and IL-10 was decreased while the expression of IFN-γ and IL-2 was increased in CD4+ T-cells collected from the EOLP group. In addition, the presence of miR-1231, miR-31 and miR-647 all obviously inhibited the expression of circ_003912 in THP-1 cells, and circ_003912 was validated to sponge the expression of these miRNAs. In addition, FOXP3 mRNA was proved to be targeted by miR-1231, miR-31 and miR-647.

Treg plays essential roles in the defense against tissue injuries caused by inflammation (Yang et al. 2008). Consequently, in OLP patients, the existence of a substantial number of FoxP3+ cells in tissue infiltrate could suggest the presence of substantial immunosuppression, although it is doubtful that FoxP3+ cells can effectually prevent the magnitude of infiltration observed in OLP. In fact, a substantial number of FoxP3+ T cells were found to produce IFN-c, suggesting that OLP is a chronic disease despite the presence of FoxP3+CD4+ T cells (Schreurs et al. 2016). A recent study suggested that FOXP3 activates the expression of miR-146 to prevent the activation of NF-κB by suppressing the expression of Irak1 as well as Traf6, causing the apoptosis of tumor cells and suggesting the presence of a FOXP3-miR-146-NF-κB pathway in the pathogenesis of prostate cancer (Liu et al. 2015).

Acting as a suppressor of differentiation of Th1 cells through targeting PRKCε, miR-146a regulates the Th1 polarization of CD4+ T cells in human (Mohnle et al. 2015). Elevated miR-146a expression in the epithelial cells of lung alveoli can also suppress the synthesis of inflammatory chemokines including RANTES and IL-8, suggesting that miR-146a might play an essential function in CD4+ T cell migration. Furthermore, miR-146a regulates IL-2 synthesis in activated T cells, implying that miR-146a can promote T cell-induced cell changes (Perry et al. 2008; Curtale et al. 2010). Another research revealed that the level of miR-146a in OLP lesions was considerably elevated compared to that in controls (Zhou et al. 2009). Various other studies have shown that enhanced expression of miR-146a in T lymphocytes was positively associated with enhanced TNF-α expression, while miR-146a can suppress the NF-κB signaling via suppressing the expression of TRAF6 as well as IRAK1 (Taganov et al. 2006; Li et al. 2010; Boldin et al. 2011; Kutty et al. 2013). Thus, it was suggested that the elevated miR-146a expression in OLP may be caused by the elevated expression of TNF-α. In this study, we found that after the THP-1 cells were transfected with circ_003912, the expression levels of circ_003912, FOXP3 mRNA, FOXP3 protein, miR-146a, IL-4 and IL-10 were up-regulated while the expression levels of miR-1231, miR-31, miR-647, TRAF6 mRNA, TRAF6 protein, IL-4 and IL-10 were down-regulated. Circ_003912 also promoted the proliferation of THP-1 cells. On the contrary, when THP-1 were transfected with circ_003912 siRNA, opposite results were obtained. However, several previous studies reported that FOXP3 could suppress cell migration and proliferation (Liu et al. 2009; Zuo et al. 2007a,2007b; Zhang and Sun 2010; Tan et al. 2014), this controversial situation may be contributed to the difference cell types investigated, and the function of a specific molecular could be different in different cell types.

TRAF6 has been proved to be a miR-146a target in diabetes and lupus nephritis (Zheng et al. 2017; Zhuang et al. 2017). In addition, the miR-146a overexpression in HaCaT cells induced by LPS could suppress the expression of TRAF6, further confirming the regulatory relationship between TRAF6 and miR-146a. It was formerly reported that TRAF6 was substantially downregulated in OLP, indicating an essential role of TRAF6 in the pathogenesis of OLP (Wang et al. 2018). Studies have shown that TRAF6 expression is needed for Treg development, indicating that the activation of NF-jB is regulated by TRAF6 in the differentiation of Treg lineage (Chiffoleau et al. 2003; King et al. 2006). Nevertheless, other studies have suggested that TRAF6 is not crucial for the activation of NF-jB pathway in CD4+ T cells (King et al. 2006). Hence, future work is needed to identify the receptors in thymocytes upstream of TRAF6 (Shimo et al. 2011).

However, the results of our study were limited. This study was mainly an observation study, and we will perform more functional study in the future. Also, more comprehensive investigations were necessary to study more potential factors and network.

## Conclusion

In this study, we investigated the molecular mechanisms underlying the pathogenesis of EOLP which involved the functioning of circ_003912 (Additional file 1: Fig. 1). We first demonstrated that circ_003912 was up-regulated in CD4+ T-cells of the EOLP group. And miRNAs including miR-1231, miR-31 and miR-647 were sponged by circ_003912 and down-regulated in CD4+ T cells of the EOLP group, which subsequently up-regulated the expression of FOXP3 and miR-146a, and resulted in the inhibition of NF-kB to promote the proliferation of CD 4+ T-cells.

## Supplementary Information

**Additional file 1: Figure 1.** Flowchart of the molecular mechanisms underlying the pathogenesis of EOLP which involved the functioning of circ_003912.

## Data Availability

The data that support the findings of this study are available from the corresponding author upon reasonable request.
