# Supplementary figures and images for: Deregulation of circ_003912 contributes to pathogenesis of erosive oral lichen planus by via sponging microRNA-123, -647 and -31 and upregulating FOXP3
Source: Mol Med. 2021 Oct 20;27:132. doi: 10.1186/s10020-021-00382-4 (PMC8527710; doi:10.1186/s10020-021-00382-4)

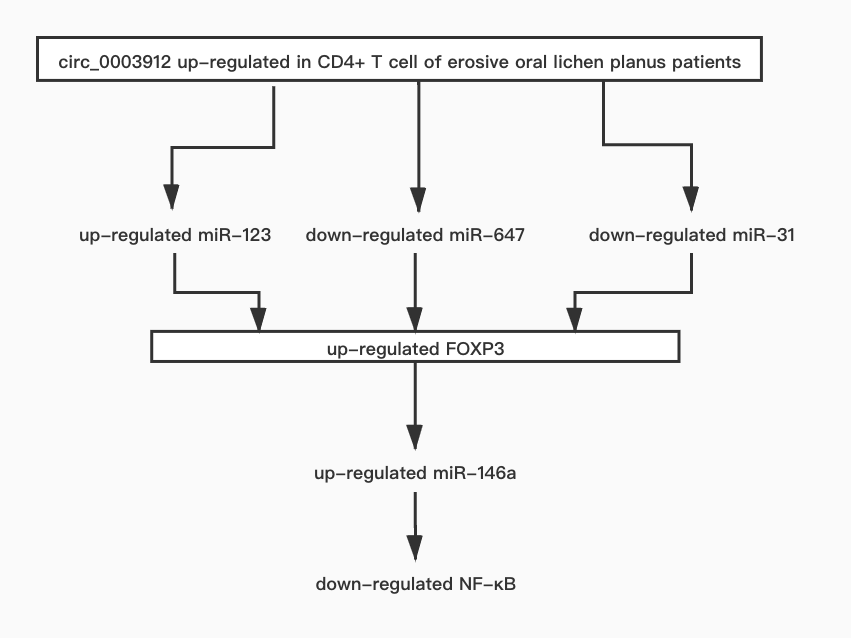

Supplement: Supplementary file 1 — Additional file 1: Figure 1. Flowchart of the molecular mechanisms underlying the pathogenesis of EOLP which involved the functioning of circ_003912. [file 10020_2021_382_MOESM1_ESM.jpeg]
